# A Novel Allosteric Inhibitor Targets PLK1 in Triple Negative Breast Cancer Cells

**DOI:** 10.3390/biom12040531

**Published:** 2022-03-31

**Authors:** Jankiben R. Patel, Prasad Thangavelu, Renee M. Terrell, Bridg’ette Israel, Arindam Basu Sarkar, A. Michael Davidson, Kun Zhang, Rahul Khupse, Syreeta L. Tilghman

**Affiliations:** 1 Division of Pharmaceutical Sciences, College of Pharmacy and Pharmaceutical Sciences, Institutes of Public Health, Florida A&M University, 1415 S. Martin L. King Jr. Blvd, Tallahassee, FL 32307, USA; jankiben1.patel@famu.edu (J.R.P.); renee1.terrell@famu.edu (R.M.T.); bridgette.israel@famu.edu (B.I.); michael.davidson@famu.edu (A.M.D.); 2 College of Pharmacy, University of Findlay, 1000 N Main St., Findlay, OH 45840, USA; prasad.thangavelu@gmail.com (P.T.); basusakar@findlay.edu (A.B.S.); 3 Department of Computer Science, Division of Mathematical and Physical Sciences, College of Arts and Sciences, Xavier University of Louisiana, New Orleans, LA 70125, USA; kzhang@xula.edu

**Keywords:** polo-like kinase 1, triple-negative breast cancer, mammospheres, allosteric inhibitor

## Abstract

While Polo-like kinase 1 (PLK1) inhibitors have shown promise in clinical settings for treating triple-negative breast cancer tumors and other solid tumors, they are limited by their ability to bind non-selectively to the ATP kinase domain. Therefore, we sought to develop a PLK1 allosteric inhibitor targeting the PLK1 T-loop (a switch responsible for activation) and evaluate its effects in triple-negative breast cancer cells. A novel compound, RK-10, was developed based on an in silico model, and its effects on specificity, viability, migration, and cell cycle regulation in MCF-10A and MDA-MB 231 cells were evaluated. When MDA-MB 231 cells were treated with 0–50 µg/mL RK-10, phospho-PLK1 (Thr-210) was decreased in cells cultured adherently and cells cultured as mammospheres. RK-10 significantly inhibited viability after 24 h; however, by 48 h, 25–50 µM RK-10 caused >50% reduction. RK-10 attenuated wound healing by up to 99.7% and caused S and G2/M cell cycle arrest, which was associated with increased p21 expression. We developed a novel allosteric inhibitor which mediates anti-proliferative and anti-migratory properties through targeting phospho-PLK1 (Thr-210) in mammospheres and causing S phase and G2/M cell cycle arrest. Further development of PLK1 allosteric inhibitors may be a promising approach for TNBC treatment.

## 1. Introduction

While a few therapeutic advances in the treatment of triple negative breast cancer (TNBC) have been made, there are still major limitations on current clinical therapy. These limitations include not only debilitating off-target effects attributed to non-selective action of chemotherapy drugs but also recurrence due to the survival and outgrowth of de novo chemo- and radiation-resistant cancer stem cells (CSCs). Therefore, there remains an urgent need to develop effective treatment strategies to combat these highly aggressive TNBC tumors. Previously, a genome-scale shRNA (short hairpin RNA) screen using the SUM series of human basal breast cancer cell lines was conducted and polo-like kinase 1 (PLK1), which is essential for survival, was identified, thus underscoring its relevance in breast cancer growth [1]. In addition, the Conceptions of Learning and Teaching (COLT) analysis of essential genes in 29 breast cancer lines identified PLK1 as a hit in 27 (93%) of these lines, including MDA-MB-468 and MDA-MB-231 TNBC cells [1]. These findings, along with other clinical findings, implicate PLK1 as a promising potential target for TNBC treatment. To this end, numerous PLK1 inhibitors have been developed such as volasertib [2] and onvasertib [3] and are currently in clinical trials for solid tumors. However, some agents are non-selective ATP-competitive inhibitors with the potential to cause toxicity due to interference with other kinases.

The current PLK1 inhibitors in clinical trials are primarily designed to target the ATP binding pocket of the N-terminal kinase domain of activated PLK1. Since our compounds do not compete with high concentrations of ATP, selectivity for PLK can be achieved with relatively low non-toxic doses, thereby avoiding the off-target side effects associated with pan kinase inhibition. Allosteric targeting of the inactive state-PLK1 provides superior selectivity, thus circumventing the development of resistance due to mutations in the ATP-binding domain. However, the development of resistance due to mutations in the allosteric site may remain a possibility.

It has been demonstrated that PLK1 activity is controlled by a balanced methylation and phosphorylation switch. The methyltransferase, G9a, monomethylates PLK1 at Lys-209, which antagonizes phosphorylation at Thr-210 to inhibit PLK1 activity; interestingly, during DNA damage repair, the interaction of G9a and PLK1 is enhanced [4]. This being so, there is a well-established precedence that phosphorylation of Thr-210 in the PLK1-T-loop is a critical switch, which controls PLK1 activation during the cell cycle and plays an important role in DNA damage repair. Thus, targeting Thr-210, the master switch in the activation loop, leads to allosteric inhibition of PLK1 activation and poses an attractive strategy for TNBC-selective anticancer agents. Here, we sought to use an in silico approach to develop a novel PLK1 inhibitor. Unlike the current PLK1 inhibitors, which bind to the ATP kinase domain, we developed a compound that targets the T-loop activation-switch region containing Thr-210 of PLK1 and evaluated its effects on the biology of TNBC cells.

## 2. Materials and Methods

### 2.1. Kaplan–Meier (KM) Survival Analysis

The application of a KM plot has been described in detail previously [5,6,7]. Briefly, KM plots were obtained using the KM Plotter web-based curator (kmplot.com/analysis), which surveys public microarray repositories for relapse-free and overall survival among patients with breast, lung, ovarian, or gastric cancers. The database was accessed on 1 September 2021. The KM Plotter recognizes 54,675 individual Affymetrix probe sets and surveys expression data from 4142 breast cancer patients (as of 2014). Survival and gene expression data were derived from the GEO (Gene Expression Omnibus), TCGA (The Cancer Genome Atlas), and EGA (European Genome-phenome Atlas) databases. In order to ascertain PLK1 expression, Affymetrix probe 202240 was selected. Relapse-free survival (RFS) in the total population (4929 patients), overall survival (OS) in the total population (1879 patients), and distant metastasis-free survival (DMFS) in the total population (2767 patients) were determined.

### 2.2. Gene Ontology

Functional enrichment analysis was performed using the Database for Annotation, Visualization and Integrated Discovery (DAVID) tool [8], which is a resource consisting of an integrated biological knowledgebase and analytical tools aimed at systematically extracting biological meaning from gene/protein expression. Using this tool, Gene Ontology (GO) and Kyoto Encyclopedia of Genes and Genomes (KEGG) pathways were identified.

### 2.3. Cell Culture

In this study, two breast cell lines were utilized. The MCF-10A cells were immortalized breast epithelial cells cultured and maintained in DMEM F-12 media (Invitrogen, Waltham, MA, USA) supplemented with 5% horse serum (Gibco, Waltham, MA, USA), penicillin-streptomycin (Invitrogen, Waltham, MA, USA), epidermal growth factor (Invitrogen Waltham, MA, USA), cholera toxin (Invitrogen, Waltham, MA, USA), hydrocortisone (Invitrogen, Waltham, MA, USA), and human insulin (Sigma Aldrich, St. Louis, MO, USA). The MDA-MB 231 and MDA-MB 468 triple-negative breast cancer cells were maintained in phenol red DMEM media (Invitrogen, Waltham, MA, USA) supplemented with 5% fetal bovine serum (Invitrogen, Waltham, MA, USA), penicillin-streptomycin (Invitrogen, Waltham, MA, USA), and antimycotic-antibiotic (10,000 U/mL penicillin G sodium; 10,000 mg/mL streptomycin sulfate) (Invitrogen, Waltham, MA, USA). The cells were maintained in a tissue culture incubator in a humidified atmosphere of 5% CO_2_ and 95% air at 37 °C. All cells were purchased from the American Type Culture Collection.

### 2.4. Viability Assays

The MCF-10A, MDA-MB 231, and MDA-MB 468 cells were seeded in 96-well plates at a density of 1 × 10^3^ cells/well in a total volume of 100 mL and allowed to attach overnight. Background levels were determined by preparing blank samples, with media added to wells in the absence of cells. On the following day, cells were treated with indicated doses of RK-10 or DMSO (vehicle). Afterwards, 10 µL of resazurin dye (Sigma Aldrich, St. Louis, MO, USA) was added to each well and incubated for 4 h at 5% CO_2_ and 37 °C. Samples were agitated for 1 min and fluorescence was measured at 24 or 48 h using a Biotek Synergy H1 microplate reader (BioTek Instruments, Inc) to measure fluorescence intensity at 550 nm excitation/590 nm emission-background wavelengths. All experiments were performed with *n* ≥ 3 and a total of 3 biological replicates were performed. The proliferative activity was determined and calculated using: Proliferative activity = [Fluorescence of viable cells − Fluorescence of blank (media only)].

### 2.5. In Silico Docking Study

A molecular docking program (Auto-Dock 4.2) was used to design a new lead compound, RK-10, and analyze the interactions between the compound and PLK1. The PLK1 crystal structure (PDB ID: 3FC2) and Discovery Studio Visualizer along with Chimera were used to visualize protein–ligand interactions and MODELLER was used for homology modeling [9,10,11]. Graphical User Interface: AutoDock Tools (ADT) was used for the preparation of pdbqt files for the protein and ligand and for the grid box creation. AutoGrid was employed for the preparation of the grid map, and the grid size was set to 60 × 60 × 60 xyz points with grid spacing at 0.375 A. During docking, both the protein and ligand were considered as rigid and the outcomes of docking with 1.0 A in positional root-mean-square deviation (RMSD) were clustered together. The docking pose with the most favorable parameters (i.e., lowest energy or binding affinity) was aligned with protein structure and further analyzed using the BIOVIA discovery studio visualizer.

### 2.6. Western Blotting Analysis

Adherent cells or cells cultured as mammospheres were treated with 0, 10, 25, or 50 µM of RK-10 for 24 h or 48 h and homogenized in cold RIPA buffer supplemented with 2× protease and phosphatase inhibitors (ThermoFisher Scientific, Waltham, MA, USA). The supernatant was incubated with Laemmli protein sample buffer (Bio-Rad, Hercules, CA, USA) at 70 °C for 10 min. About 75 µg of denatured protein was separated on 4–20% Mini-PROTEAN^®^ TGX™ Precast Protein Gel (Bio-Rad, Hercules, CA, USA) and transferred to PVDF membranes. All blots were blocked for 1 h with 5% Bovine Serum Albumin (BSA) in Phosphate-Buffered Saline, 0.1% Tween (PBS-T) buffer. Following incubation with anti-p21 antibody (Cell Signaling Technology, Danvers, MA, USA), anti-PLK1 antibody (Cell Signaling Technology, Danvers, MA, USA), or anti-phospho PLK1 Thr-210 antibody (Cell Signaling Technology, Danvers, MA, USA) and secondary anti-rabbit antibodies (Cell Signaling Technology, Danvers, MA, USA), the blots were visualized using a ChemiDoc XRS imaging system (BioRad, Hercules, CA, USA) and detected with the Clarity Max Western ECL Substrate (BioRad, Hercules, CA, USA). The exposure time was automatically detected by the imaging system. The protein bands were analyzed using Image Lab software (BioRad, Hercules, CA, USA). Arbitrary densitometry units were quantified and expressed as means ± standard deviations. PLK1 and phospho-PLK1 protein expression was normalized to the GADPH housekeeping protein bands. All experiments were performed with *n* ≥ 3 and a total of 3 biological replicates were performed.

### 2.7. Kinase Antibody Arrays

To compare the impact of RK-10 activation/phosphorylation in the MDA-MB 231 cells, the Human MAPK Phosphorylation Antibody Array (ab211061, Abcam, Cambridge, MA, USA) and Human Apoptosis Antibody Array (ab134001, Abcam, Cambridge, MA, USA) were evaluated. The antibody arrays consisted of nitrocellulose membranes containing antibodies spotted in duplicate, including positive and negative controls and a blank. Cell lysates were prepared from the MDA-MB 231 cells using Cell Lysis Buffer supplemented with Phosphatase Inhibitor and Protease Inhibitor Cocktail and stored at −80 °C until use. For each cell lysate, 900 μg of total protein was determined by the Bradford Assay (BioRad, Hercules, CA, USA) and incubated in 2 mL of 1× Blocking Buffer at room temperature for 30 min. The antibody array membranes were washed and subsequently incubated with Detection Antibody Cocktail overnight at 4 °C to detect proteins. After washing and incubation with HRP-streptavidin, the membranes were subjected to visualization with a chemiluminescence-based detection method.

### 2.8. Wound Healing Assay

The wound healing assay was performed as previously described [12]. Briefly, an Ibidi 2-well culture-insert (Ibidi, Gräfelfing, Germany) was inserted in a 6-well culture plate, then MCF-10A and MDA-MB 231 cells were seeded at a density of 1.5 × 10^5^ cells/well and grown until 100% confluent. The insert was removed to create a cell-free gap and serum-free medium was added. Each scratch was immediately imaged (at 0 h) 3 times at different locations using the 5× objective on a Zeiss AX10 fluorescence microscope. Each well was treated with DMSO control, 10 μM, 25 μM, or 50 μM RK-10 and incubated for 24 h at 37 °C with 5% CO_2_. Images were captured after 24h and 48 h and the cell-free gap area was measured using Olympus CellSens Standard 1.16 software. The percent wound area was analyzed as follows: Percent Wound Area = 100-Percent Wound Closure, where percent wound closure = [(initial wound area-wound area)/initial wound area] × 100%. All experiments were performed with *n* ≥ 3 and a total of 3 biological replicates were performed.

### 2.9. Mammosphere Formation Assay

The mammosphere formation assay was performed as previously described [12]. Briefly, the MCF-10A and MDA-MB 231 cells were grown to 80–90% confluence, and after medium was removed, cells were rinsed twice with Hank’s Balanced Salt Solution (HBSS) (Stemcell Technologies, Vancouver, BC, Canada) to remove residual culture media. Cells were gently scraped and resuspended in 10 mL of MammoCult™ media (Stemcell Technologies, Vancouver, BC, Canada). Afterwards, cells were centrifuged at 500× *g* for 3 min at room temperature. The supernatant was discarded and the pellet was resuspended into a single-cell suspension in 2 mL of MammoCult™ medium. Cell concentration and viability were determined with Trypan Blue exclusion. Cells were enumerated and 100,000 cells were seeded in ultra-low adherent plates. The cultures were incubated in a 5% CO_2_, humidified incubator at 37 °C for 3 days and spheres were allowed to form. On the following day, the spheres were treated with vehicle control, 10, 25, or 50 µM RK-10. On day 7, the cells were harvested and spheres greater than 60 µm were counted and recorded. Mammosphere formation was identified by light microscopy and then harvested as indicated in Western blot analysis. All experiments were performed with *n* ≥ 3 and a minimum of 3 biological replicates were performed.

### 2.10. Cell Cycle Analysis

MDA-MB-231 cells were seeded at a density of 1.5 × 10^6^ cells in a 75 cm^2^ flask and allowed to grow to 70–80% confluence. The cells were washed twice with PBS. Afterwards they were treated with DMSO control, 10 μM, 25 μM, or 50 μM RK-10 and incubated for 48 h at 37 °C with 5% CO_2_. After 48 h treatment, the media were removed, cells were trypsinized using 0.25% Trypsin-EDTA, centrifuged at 300× *g* for 8–10 min and the supernatant was removed. Cells were fixed by adding 5 mL of ice-cold 70% *v*/*v* absolute ethanol dropwise and stored at 4 °C overnight. The samples were centrifuged at 300× *g* for 8–10 min and the supernatant was removed, leaving approximately 200 μL for re-suspended cells. The pellets were re-suspended in 1× PBS, centrifuged at 300× *g* for 8–10 min, and the supernatant was removed. Cells were stained with 1 mL of Propidium Iodide (PI) solution (100 μL PI stock, 25 mg RNAse A, 5–10 mg glucose or dextrose, and 10 mL 1× PBS (without Mg^2+^ and Ca^2^)) and incubated for 1 h at 37 °C. Subsequent analysis of DNA distribution in cell cycle phases was performed using a BD FACS Calibur flow cytometer (FACS caliber, BD Biosciences, San Jose, CA, USA) in replicates. CellQuest Pro software was used to determine the percentage of different cell cycle phase distribution (G0/G1, S, and G2/M phases) and the percentage of cells in each phase was quantified using ModFit LT 3.2.1 Software (Verity software house Inc, Topsham, ME, USA).

### 2.11. Statistical Analysis

Results are expressed as the mean units ± standard errors of the means (SEMs) (**** *p <* 0.0001, *** *p* < 0.001, ** *p* < 0.01, * *p* < 0.05) using the Graph Pad Prism V.6 software program.

## 3. Results

### 3.1. High PKL1 Expression Is Positively Correlated with Decreased Survival among Breast Cancer Patients

**Figure 1:**
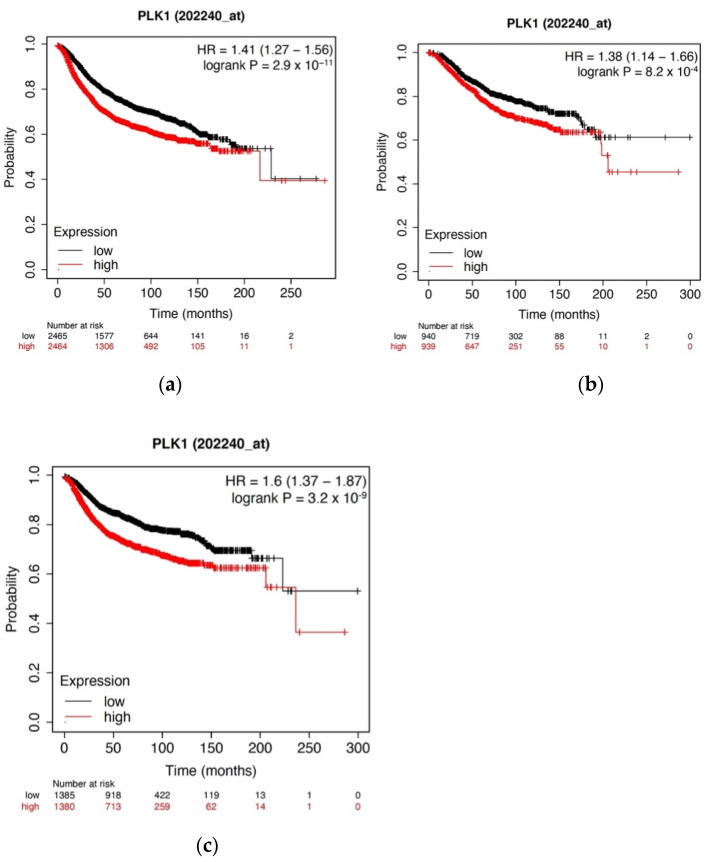
High PKL1 expression is positively associated with decreased RFS, OS, and DMFS in breast cancer patients. Kaplan–Meier plots of (**a**) RFS, (**b**) OS, and (**c**) DMFS based on PLK1 expression in breast cancer patients. Using Kaplan–Meier Plotter, publicly available microarray repositories for breast cancer were interrogated to determine whether PLK1 expression was associated with different survival rates among breast cancer patients. Hazard ratio (HR) and log-rank *p*-values are shown. Low PLK1 expression (below median) is noted in black; high PLK1 expression (above median) is noted in red.

PLK1 is overexpressed in many cancers and serves as a well-validated target for new drugs in pre-clinical and clinical studies. To validate the role of PLK1 as a negative prognostic indicator in breast cancer, we used KM Plotter to interrogate publicly available microarray repositories for breast cancer patients. We examined whether PLK1 expression was associated with differences in RFS and found that high PLK1 expression was associated with a substantial decrease in RFS (*p* = 2.9 × 10^−11^) (Figure 1). Additionally, high expression of PLK1 was also associated with decreased OS (*p* = 0.00082) and DMFS (*p* = 3.2 × 10^−9^), indicating that high PLK1 expression is associated with more relapses, worse survival, and more distant metastasis. This suggests the preclinical validity of PLK1 as a potential molecular target for breast cancer. Functional enrichment analyses were performed utilizing the NIH DAVID tool (https://david.ncifcrf.gov/) (Supplementary Table S1) which was accessed on 17 March 2022, and PLK1 was found to be highly involved in 189 highly specific biological processes, including regulation of kinase activity and negative regulation of cyclin-dependent protein kinase activity and four KEGG pathways, including hsa04110: Cell Cycle and others. PLK1 was also found to interact with five protein features, including but not limited to the protein kinase-like domain and the protein kinase ATP binding site and catalytic site. Based on these results, we chose to synthesize a novel, allosteric PLK1 inhibitor.

### 3.2. RK-10 Targets Critical Amino Acids Required for PLK1 Activation

**Figure 2:**
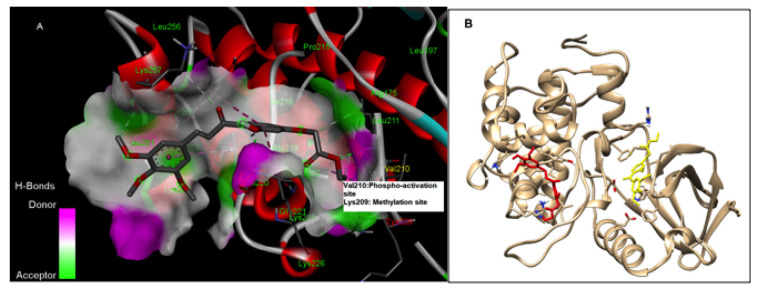
Binding interaction of the RK-10 compound. Using the computer-aided docking program Autodock 4.2, a model for binding of RK-10 with PLK1 (PDBID:3FC2) was created. The RK-10 compound binds to the allosteric site interacting with Lys-209 and Val-210. The Thr-210 residue in PLK1 is the “master switch” and a critical site residue, which becomes activated by phosphorylation, while methylation of Lys-209 by G9a prevents phosphorylation. In the crystal structure, the regulatory phosphorylation site, Thr-210, was mutated to Val-210 to reduce conformational heterogeneity. (**A**) Hydrogen binding interaction map of RK-10 with the Val-210 phospho-activation site, which is highlighted in yellow. (**B**) The docking model for the comparison of the binding mode of RK10 (red) with mutated Lys-209 Met (yellow). The mutation of Lys-209 to Met (a methyl-mimetic residue) inhibits the binding of RK-10 to the original site.

Current PLK1 inhibitors are primarily designed to target the ATP-binding pocket of the N-terminal kinase domain of activated PLK [13]. Methyltransferases such as G9a regulate the activity of PLK1 by monomethylation at Lys-209, which prevents the phosphorylation of Thr-210 and inhibits PLK1 activation. During DNA damage repair, the interaction of G9a and PLK1 is enhanced [4]. Given this, there is a well-established precedence that phosphorylation of Thr-210 in the PLK1-T-loop is a critical switch which controls PLK1 activation during the cell cycle and plays an important role in DNA damage repair. Thus, targeting Thr-210, the master switch in the activation loop, leads to allosteric inhibition of PLK1 activation and poses an attractive strategy for TNBC-selective anticancer agents. Therefore, we established a computer-aided docking model for the screening of various RK-10 compounds for PLK1 inhibition. This model indicated that RK-10 interacts with the critical amino acid residues near the DFG-motif, including Thr-210, which is required for the phosphorylation and activation of PLK1 during mitosis (Figure 2A). Based on the binding affinities and the selectivity of the compounds for the allosteric site near Thr-210 of PLK1, the specific amino acid residue interactions that are critical for PLK1 Thr-210-phosphorylation were targeted. When Lys-209 was substituted for Met at position 209 (i.e., methyl-mimetic) in PLK1, the RK-10 binding site was completely altered, as indicated by the docking model (Figure 2B). Thus, the binding of RK-10 to the inactive form of PLK1 may be dependent on the methylation status of Lys-209. The designed compound was synthesized using standard medicinal chemistry methods.

### 3.3. Novel Allosteric Inhibitor Decreases the Viability of TNBC Cells

**Figure 3:**
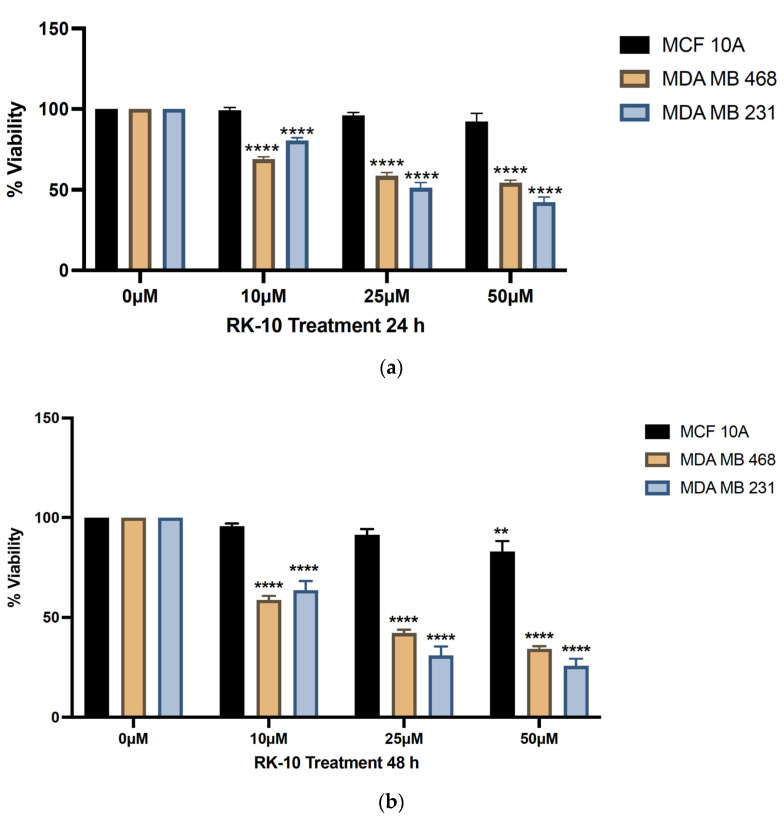
RK-10 inhibits the proliferation of triple-negative breast cancer cells. The effect of RK-10 on the proliferation of MCF-10A, MDA-MB 231, and MDA-MB 468 cells was evaluated after (**a**) 24 and (**b**) 48 h. Student’s *t*-tests were performed and treatments were compared to the DMSO control. Results are expressed as the means ± SDs (****p < 0.0001, ** *p* < 0.01) and the data are representative, from one of at least three independent experiments.

Given that previous reports demonstrated the anti-proliferative effects of PLK1 inhibitors on MDA-MB-231 cells [14], we chose to examine whether the RK-10 compound inhibited the proliferation of MDA-MB-231 and MDA-MB 468 cells. The results demonstrated that as early as 24 h, the 10 µM dose of RK-10 modestly inhibited cell proliferation. When the MDA-MB 231 cells were treated with 25–50 µM RK-10, the viability was inhibited by ≥50%, while the MDA-MB 468 cells were slightly less sensitive. The MCF-10A normal breast epithelial cells were unaltered by RK-10 treatment (Figure 3). Interestingly, when both TNBC cell lines were treated with RK-10 for 48 h, the inhibitory effect was more pronounced, with concentrations as low as 10 µM causing more than 40% inhibition, indicating that RK-10 may be a promising agent for TNBC.

### 3.4. RK-10 Inhibits Phosphorylated PLK1 Protein Expression

**Figure 4:**
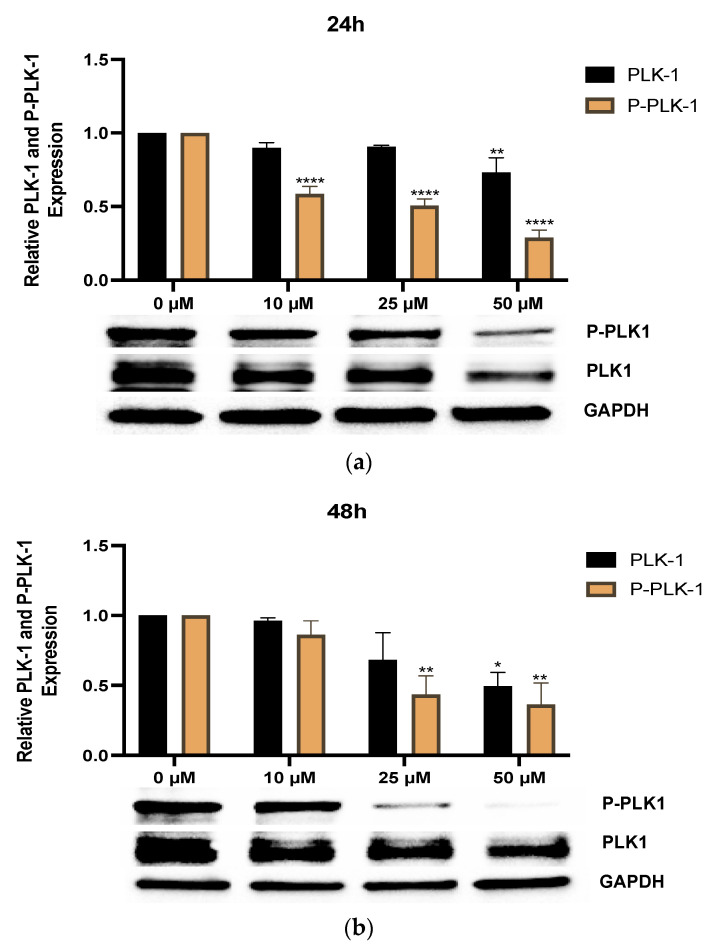
RK-10 decreases PLK1 expression. PLK1 and phosphorylated PLK1 protein expression was measured in MDA-MB 231 cells. Cells were treated with 0, 10, 25, and 50 µM of RK-10 for (**a**) 24 h and (**b**) 48 h and assayed by immunoblotting to examine the expression levels of PLK1, phosphorylated PLK1, and GAPDH (loading control). Student’s *t*-tests were performed and treatments were compared to the DMSO control. Results are expressed as the means ± SDs (**** *p* < 0.0001, **p < 0.01, * *p* < 0.05), and data are representative, from one of at least three independent experiments.

Since RK-10 inhibited cell proliferation, we chose to validate the specificity of RK-10 to target phospho-PLK1 (Thr-210) protein expression in TNBC. When cells were treated with 10 µM and 25 µM RK-10 for 24 h, the total levels of PLK-1 were relatively unaffected (Figure 4a); however, there was a dose-dependent decrease in the phosphorylation of PLK1, indicating that RK-10 selectively inhibits the activation of PLK1 at Thr-210. This trend was maintained at 48 h (Figure 4b). Since 50 µM RK-10 reduced both phospho-PLK1 and total PLK1, we compared the ratio of phospho-PLK1 to total PLK1 to determine whether the change in phospho-PLK1 was significant (Supplementary Figure S1). At 24 h, 50 µM RK-10 induced a significant decrease in phospho-PLK1 but not at 48 h. We also measured the impact of RK-10 on PLK1 and phospho-PLK1 expression in normal MCF-10A cells, and there was not a statistically significant difference in the protein expression between the RK-10-treated group and the controls (Supplementary Figure S2). It was equally important to measure the selectivity of RK-10 to target additional kinases. Therefore, a membrane-based antibody array was used to measure the impact of RK-10 treatment on the levels of protein kinase phosphorylation. In this setting, RK-10 did not significantly impact the phosphorylation status of the tested proteins (i.e., phospho-AKT Ser-473, ERK1/ERK2, phospho-GSKa Ser-21, phospho-GSKb Ser-9, phospho-JNK Thr 183, phospho-MEK Ser-217/221, phospho-MKK3 Ser-189, phospho-MKK6 Ser-360, phospho-MSK2 Ser-360, phospho-mTOR, phospho-p38 Thr-180/Tyr-182, phospho-p53 Ser-15, P70S6K, phospho- RSK1 Ser-380, and phospho-RSK2 Ser-386) in MDA-MB 231 cells (Supplementary Figure S3). Our observations confirmed that RK-10 inhibited the intended target, phospho-PLK1 (Thr-210), which is likely responsible for the growth inhibitory effects in TNBC shown in Figure 3.

### 3.5. RK-10 Attenuates Cell Motility

**Figure 5:**
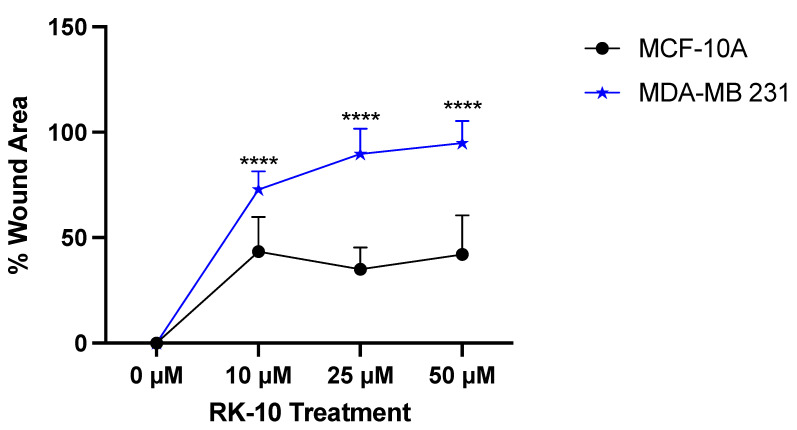
RK-10 attenuates wound healing. MCF-10A and MDA-MB 231 cells were treated with RK-10 and migration was measured using the wound healing assay. The wound distance was measured after 24 h and the graph represents the percentage of the wound area. Student’s *t*-tests were performed and treatments were compared to the DMSO control. Results are expressed as the means ± SDs (**** *p* < 0.0001), and data are representative, from one of at least three independent experiments.

Since previous studies have implicated that PLK1 leads to the epithelial-to-mesenchymal transition (EMT) and affects invasion and metastasis [15], we were interested in examining whether RK-10 altered cell motility. To accomplish this, wound healing assays were conducted whereby a wound was made and the cells were exposed to increasing doses of RK-10. After 24h and 48 h, the area where the wound was made was measured and, at all doses, RK-10 selectively prevented wound closure in the MDA-MB 231 cells, which persisted up to 48 h (Figure 5). At 24 h, the 25 µM and 50 µM dose of RK-10 prevented wound healing by 97.3% and 99.8%, respectively. While all doses of RK-10 altered wound healing in the MCF-10A cells, the percent wound closure was dramatically lower compared to the MDA-MB 231 cells (Supplementary Figure S4).

### 3.6. RK-10 Selectively Retards Activation of PLK1 in TNBC Mammospheres

**Figure 6:**
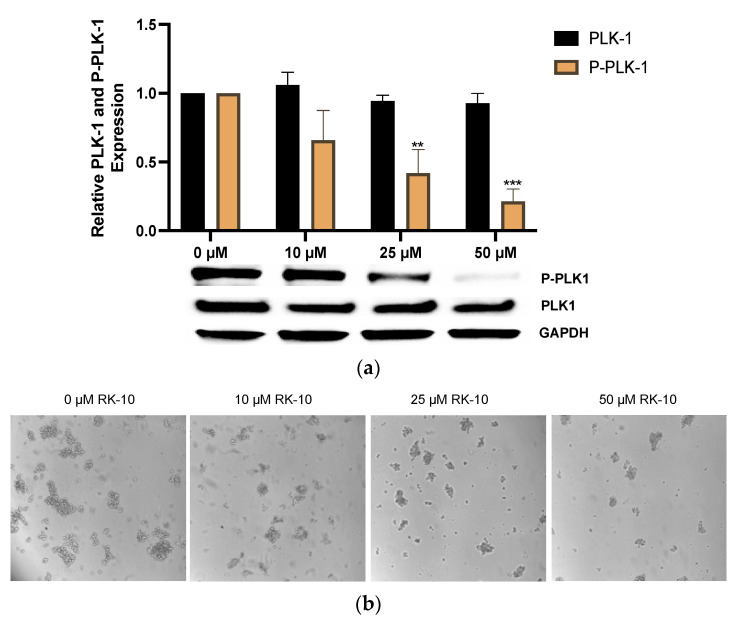
RK-10 inhibits activated PLK1 in MDA-MB 231 mammospheres. (**a**) MDA-MB 231 cells were cultured as mammospheres and treated with 0, 10, 25, and 50 µM of RK-10 for 24 h. Afterwards, the cells were assayed by immunoblotting to examine the expression levels of phosphorylated PLK1 and GAPDH (loading control). (**b**) Representative microscopic images of RK-10-treated MDA-MB 231 mammopsheres. Student’s *t*-tests were performed, and treatments were compared to the DMSO control. Results are expressed as the means ± SDs (*** *p* < 0.001, ** *p* < 0.01), and data are representative, from one of at least three independent experiments.

Recent studies have shown that a small subpopulation of highly tumorigenic cancer stem cells (i.e., CSCs or tumor-initiating cells) that are de novo radiation- and chemo-resistant are involved in relapse and metastasis and implicated in EMT in TNBCs [16]. The proliferative and invasive capacity of CSCs mediates metastasis, and, due to their pluripotent nature, they can undergo multi-lineage differentiation and repopulate a tumor. Previous gene expression profiling studies using small-molecule kinase inhibitors demonstrated that PLK1 inhibition may lead to the elimination of CSCs in a range of tumors [17,18]. It is well established that the mammosphere formation assay is a surrogate indicator for the presence of CSCs [19,20,21]. This independent finding, along with our observation of the antiproliferative activity of RK-10 activity in TNBCs and decreased cell motility, prompted us to test whether RK-10 could alter activated PLK1 (Thr-210) in MDA-MB-231 mammospheres. As such, we cultured MDA-MB-231 cells as mammospheres as previously described [22], then treated them with RK-10 at various concentrations and measured phospho-PLK1 (Thr-210) expression. These results demonstrated an RK-10 induced decrease in phospho-PLK1 (Thr-210) expression in the mammospheres (Figure 6), which indicates the potential of this agent to selectively target this kinase in an aggressive subpopulation of TNBC cells. These findings indicate that RK-10 exhibits anti-proliferative activity within the heterogeneous MDA-MB 231 cell population as well as within the aggressive CSC subpopulation, making it a promising lead molecule that may be useful for the elimination of both cellular populations.

### 3.7. RK-10 Induces S Phase and G2/M Cell Cycle Arrest

**Figure 7:**
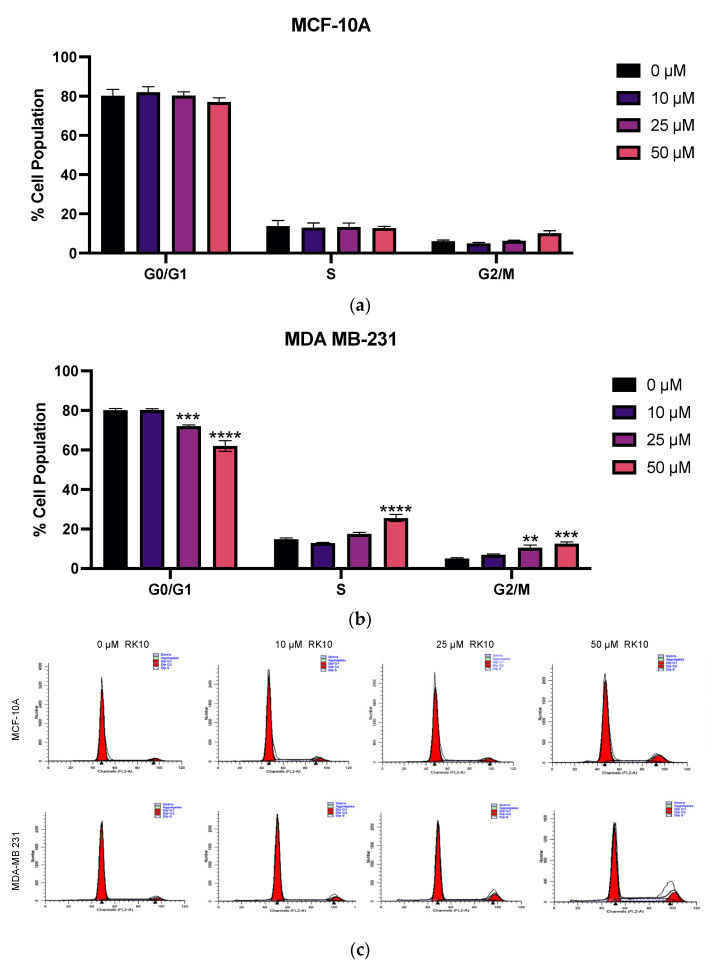
RK-10 induces S and G2/M cell cycle arrest. (**a**) MCF-10A and (**b**) MDA-MB 231 cells were treated with 0, 10, 25, and 50 µM of RK-10 for 48 h. Afterwards, the cells were fixed, stained, and flow cytometry was performed. (**c**) Representative histograms of the flow cytometry profiles monitoring cell cycle progression as determined by the distribution of DNA content. Student’s *t*-tests were performed, and treatments were compared to the DMSO control. Results are expressed as the means ± SDs (**** *p* < 0.0001, *** *p* < 0.001, ** *p* < 0.01), and data are representative, from one of at least three independent experiments.

PLK1 is a serine/threonine kinase, which is highly expressed in the G2 phase of the cell cycle, peaks in the M phase and controls the crucial transition from metaphase-to-anaphase and mitotic exit [23,24]. Since PLK1 regulates multiple steps in mitosis and predominantly affects dividing cells, we sought to examine whether RK-10 altered progression through the cell cycle. Therefore, both MCF-10A and MDA-MB 231 cells were evaluated for progression through the cell cycle following RK-10 treatment. While there was no significant change between vehicle-treated and RK-10-treated MCF-10A cells, the 25–50 µM RK-10 treatment caused fewer MDA-MB 231 cells to accumulate in G0/G1 and more cells to accumulate in the S and G2/M phase compared to the vehicle control (Figure 7).

**Figure 8:**
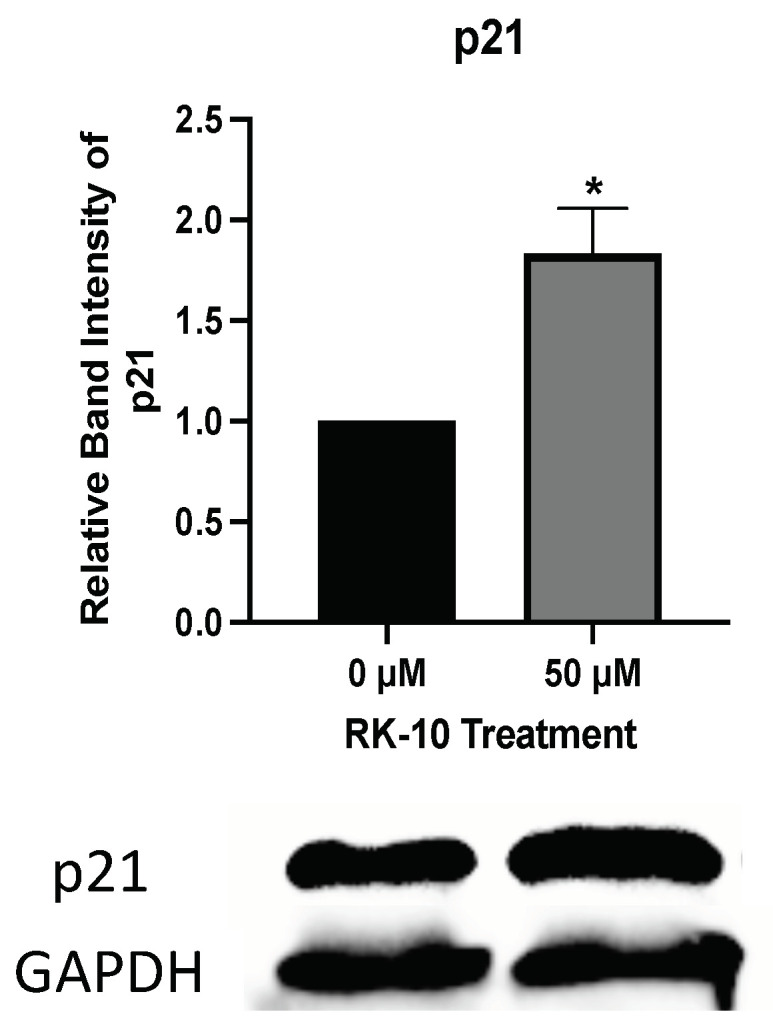
RK-10 induces p21 expression in MDA-MB 231 cells. MDA-MB 231 cells were cultured and treated with 0 or 50 µM of RK-10 for 24 h. Afterwards, the cells were assayed by immunoblotting to examine the expression levels of p21 and GAPDH (loading control). Student’s *t*-tests were performed, and treatments were compared to the DMSO control. Results are expressed as the means ± SDs (* *p* < 0.05), and data are representative, from one of at least three independent experiments.

To determine whether the RK-10-mediated cell cycle arrest was associated with cell death, we performed a focused antibody array to measure the impact of RK-10 treatment on proteins involved in apoptosis (Supplementary Figure S5). Interestingly, compared to the DMSO control, RK-10 treatment had no effect on the expression of Bad, Bax, Bcl-2, Bcl-w, Bid, Bim, caspase 3, or caspase 8. However, RK-10 induced a robust increase in p21 expression, which was confirmed by immunoblotting (Figure 8). This implies that the RK-10 anti-proliferative effects are mediated in part through cell cycle arrest induced by the cyclin-dependent kinase inhibitor, p21.

## 4. Discussion

TNBC is the most aggressive phenotype of breast cancer, representing about 15–20% of breast carcinomas, and is associated with African American ethnicity, younger age, and poor prognosis [25,26]. The major challenges of TNBC treatment include the lack of targeted therapies, higher recurrence rates, and lower survival due to metastasis. Absence of the estrogen receptor (ER), the progesterone receptor (PR), and human epidermal growth factor receptor 2 (HER2) poses a significant barrier compared to other subtypes of breast cancers. Therefore, clinical trials to explore new targets in TNBC, such as PI3K/AKT/mTOR, poly (ADP-ribose polymerase (PARP), RAS/MAPK, epidermal growth factor receptor (EGFR), androgen receptor, protein kinases, and altered metabolic pathways, are underway.

PLK1 is a promising target under clinical investigation in TNBC and several other cancers [18,27]. PLK1 is overexpressed in many cancers and serves as a well-validated target for new drugs in pre-clinical studies. This being so, we were interested to explore the clinical relevance of PLK1 to patient survival in a cohort of breast cancer patient samples and found that high levels of PLK1 were associated with worse RFS, DMFS, and OS. Additionally, using the DAIVD tool we found that PLK1 is associated with several GO terms many of which are involved in the cell cycle. Taken together this provided a strong rationale to design a novel PLK1 inhibitor. Several PLK1 inhibitors, such as volasertib and BI2536, are in clinical trial for solid tumors; they are primarily designed to target the ATP-binding pocket of the N-terminal kinase domain of activated PLK [20]. However, achieving selectivity for PLK1 over other kinases, which also have ATP binding sites, is a major limitation of this approach. The emergence of drug resistance due to mutations in the ATP-binding pocket is commonly observed with similar mechanisms and can limit the utility of first-generation PLK1 inhibitors. Therefore, newer selective agents, which can allosterically target the inactive form of PLK1, can circumvent these problems associated with the current PLK1 inhibitors [28].

Thus, we synthesized a novel allosteric PLK1 inhibitor which targets critical amino acids such as Thr-210. When Met-209 was substituted for Lys-209, RK-10 exhibited a different binding conformation. However, the significance of methylation of Lys-209 remains unclear and future mutation studies are required to address this concern. RK-10 successfully inhibited cell viability in two TNBC cell lines without substantially altering the growth of the normal MCF-10A breast epithelial cells. As this result was encouraging, we sought to validate the specificity to determine whether RK-10 could target phospho-PLK-1 (Thr-210) by performing Western blots. As early as 24 h, RK-10 induced a dose-dependent decrease in phospho-PLK-1 (Thr-210) expression, which was sustained at 48 h. While the 10 µM and 25 µM RK-10 did not cause alterations in total PLK-1 levels, when TNBC cells were treated with 50 µM RK-10, total PLK-1 levels were decreased at both time points, suggesting that the optimal concentration is less than 50 µM. Since total PLK1 levels were decreased with 50 µM RK-10 treatment, we compared the ratio of phospho-PLK1 to total PLK1 and at 24 h phospho-PLK1 was significantly inhibited but not at 48 h. Future in vitro and in vivo studies will be required to identify the optimal dose. Although 50 µM RK-10 did not lead to a significant decrease in phospho-PLK1 at 48 h, we were encouraged by the anti-migratory properties of RK-10 at all concentrations. This was critical, as PLK1 is implicated in enhanced cell motility and can drive cancer progression.

Therapy failure in TNBC is mainly due to the higher incidence of CSCs, which is responsible for recurrence and failure of chemotherapy. Given that CSCs are also capable of self-renewal, proliferation, and plasticity, we tested whether RK-10 could target PLK1 in this population. RK-10 was found to target phospho-PLK1 (Thr-210) not only in the adherent cell population but also in the CSC-enriched MDA-MB 231 mammospheres. This observation, in concert with the ability of RK-10 to prevent cellular migration, suggests that RK-10 could potentially play a role in targeting chemoresistance and/or curtail metastasis. We performed further studies to explore the mechanism responsible for the RK-10 mediated reduction in proliferation, and cell cycle analysis revealed that RK-10 induces S and G2/M phase cell cycle arrest. We measured the impact of RK-10 treatment on a panel of proteins involved in apoptosis and found that p21 expression was increased, as was further validated by immunoblotting.

## 5. Conclusions

Unlike current PLK1 inhibitors which target the ATP binding domain, our lead molecule potentially binds to the PLK1 T-loop, which is a switch responsible for its activation. Our approach has the potential to circumvent the major limitations of current PLK1 inhibitors, namely, selectivity for PLK1 over other kinases, which could avoid side effects due to inhibition of other kinases required in normal cellular process. Allosteric targeting of inactive PLK1 could potentially provide a better selectivity profile and prevent or delay the development of resistance due to mutations within the ATP-binding domain which is a frequent phenomenon in ATP-targeting kinases. Thus, RK-10 is a novel small-molecule PLK1 inhibitor that allosterically targets the master switch for activating PLK1 in TNBC. The development of such molecules is urgently needed to prolong survival and reduce relapse in TNBC patients.

## Data Availability

Not applicable.

## Supplementary Materials

The following supporting information can be downloaded at: https://www.mdpi.com/article/10.3390/biom12040531/s1, Table S1: PLK1 functional annotation, Figure S1: Phosphorylated PLK1 expression in MDA-MB 231 cells normalized to total PLK1. PLK1 and phosphorylated PLK1 protein expression were measured in MDA-MB 231 cells. Cells were treated with 0, 10, 25, and 50 µM of RK-10 for (**a**) 24 h and (**b**) 48 h and assayed by immunoblotting to examine the expression levels of PLK1, phosphorylated PLK1, and GAPDH (loading control). The histograms represent the average densitometry ratio of phosphorylated PLK1 to total PLK1. Student’s *t*-tests were performed, and treatments were compared to the DMSO control. Results are expressed as the means ± SDs (** *p* < 0.01, * *p* < 0.05), and data are representative, from one of at least three independent experiments, Figure S2: The effect of RK-10 on PLK-1 and phosphorylated PLK-1 in MCF-10A cells. MCF-10A cells were cultured and treated with 0 or 50 µM of RK-10 for 24 h. Afterwards, the cells were assayed by immunoblotting to examine the expression levels of PLK1 and phosphorylated PLK1 and GAPDH (loading control). Student’s *t*-tests were performed, and treatments were compared to the DMSO control. Results are expressed as the means ± SDs, and data are representative, from one of at least three independent experiments, Figure S3: The impact of RK-10 on MAPK kinase signaling. MDA-MB 231 cells were cultured and treated with 0 or 50 µM of RK-10 for 24 h. Graph depicts the normalized mean pixel density of protein levels of lysates prepared from the MDA-MB 231 cells using the Human MAPK Antibody Array Membrane whereby various antibodies were spotted in duplicate, including four positive and three negative controls and one blank. Array signals were analyzed using Image Lab (BioRad) software. Values from duplicate spots were averaged and plotted. Results are expressed as the mean units, Figure S4: RK-10 prevents wound healing in MDA-MB 231 triple-negative breast cancer cells. The (**a**) MCF-10A and (**b**) MDA-MB 231 cells were treated with RK-10, and migration was measured using the wound healing assay. The wound distance indicated in red was measured after 24 h and representative images are shown above, Figure S5: RK-10 induces p21 expression. MDA-MB 231 cells were cultured and treated with 0 or 50 µM of RK-10 for 24 h. Graph depicts the normalized mean pixel density of protein levels of lysates prepared from the MDA-MB 231 cells using the Human Apoptosis Antibody Array Membrane whereby various antibodies were spotted in duplicate, including four positive and three negative controls and one blank. Array signals were analyzed using Image Lab (BioRad) software. Values from duplicate spots were averaged and plotted. Results are expressed as the mean units.
