# Supplementary material for: A Novel Allosteric Inhibitor Targets PLK1 in Triple Negative Breast Cancer Cells
Source: Biomolecules. 2022 Mar 31;12(4):531. doi: 10.3390/biom12040531 (PMC9024838; doi:10.3390/biom12040531)
Supplement: Supplementary file 1 [file biomolecules-12-00531-s001.zip › biomolecules-1406181-supplementary-Figure S1-S5.pdf]

biomolecules

MDPI

Supplementary Materials

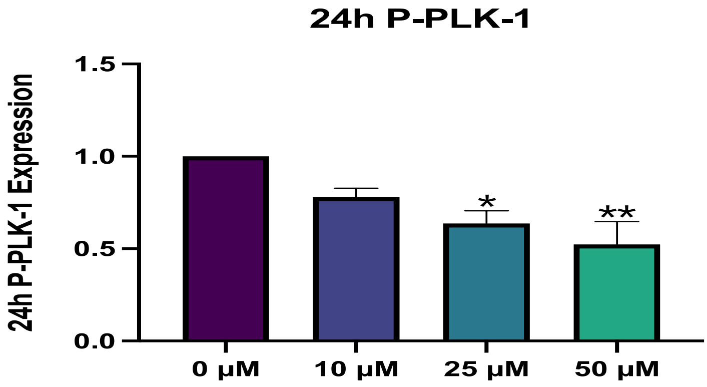

(**a**)

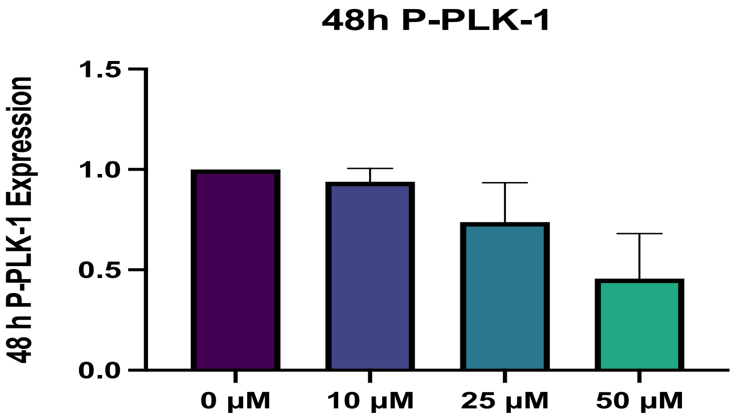

(**b**)

Figure S1. Phosphorylated PLK1 Expression in MDA-MB 231 Cells Normalized to Total PLK1. PLK1 and phosphorylated PLK1 protein expression was measured in MDA-MB 231 cells. Cells were treated with 0, 10, 25 and 50 µM of RK-10 for (**a**) 24 hours and (**b**) 48 hours and assayed by immunoblotting to examine the expression levels of PLK1, phosphorylated PLK1, and GAPDH (loading control). The histograms represent the average densitometry ratio of phosphorylated PLK1 to total PLK1. Student's *t*- tests were performed and treatment were compared to DMSO control. Results are expressed as the mean ± SD (** $p < 0.01$, * $p < 0.05$), and data are representative from one of at least three independent experiments.

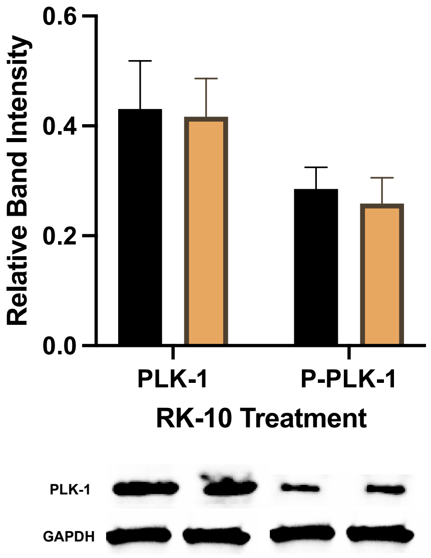

Figure S2. The Effect of RK-10 on PLK-1 and phosphorylated PLK-1 in MCF-10A Cells. MCF-10A cells were cultured and treated with 0 or 50 µM of RK-10 for 24 hours. Afterwards, the cells were assayed by immunoblotting to examine the expression levels of PLK1 and phosphorylated PLK1 and GAPDH (loading control). Student's t tests were performed, and treatments were compared to DMSO control. Results are expressed as the mean ± SD, and data are representative from one of at least three independent experiments.

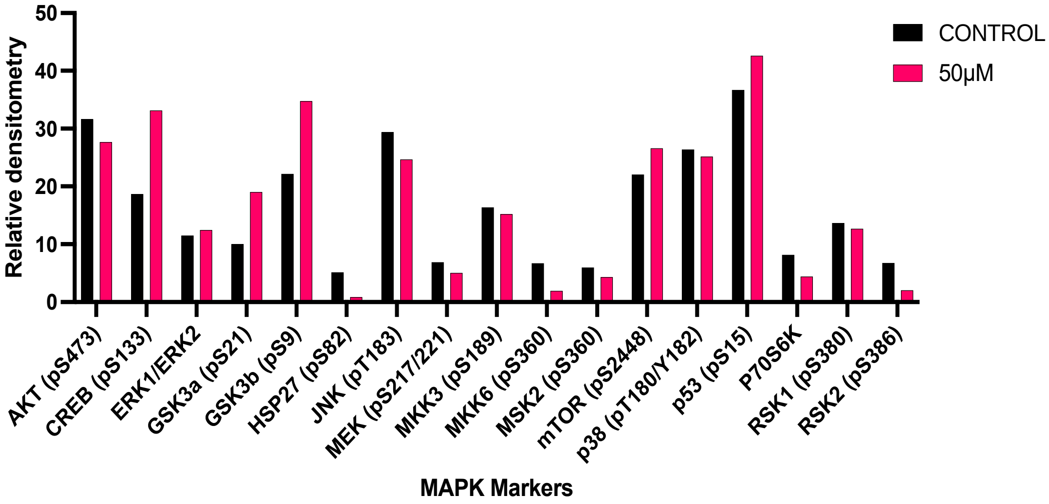

Figure S3. The Impact of RK-10 on MAPK Kinase Signaling. MDA-MB 231 cells were cultured and treated with 0 or 50 µM of RK-10 for 24 hours. Graph depicts the normalized mean pixel density of protein levels of lysates prepared from the MDA-MB 231 cells using the Human MAPK Antibody Array Membrane where various antibodies were spotted in duplicate, including 4 positive and 3 negative controls and 1 blank, Array signals were analyzed using Image Lab (BioRad) software. Values from duplicate spots were averaged and plotted. Results are expressed as the mean unit.

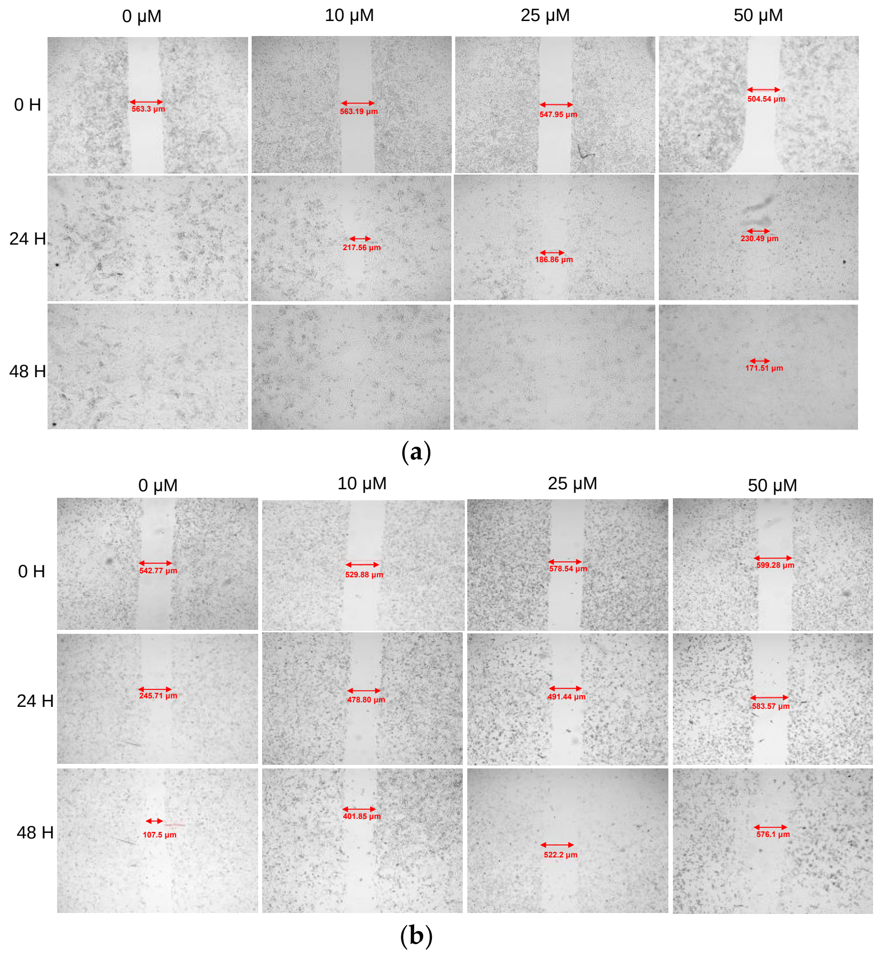

Figure S4. RK-10 Prevents Wound Healing in MDA-MB 231 Triple Negative Breast Cancer Cells. The (**a**) MCF-10A and (**b**) MDA-MB 231 cells were treated with RK-10 and migration was measured using the wound healing assay. The wound distance indicated in red was measured after 24 hours and representative images are shown above.

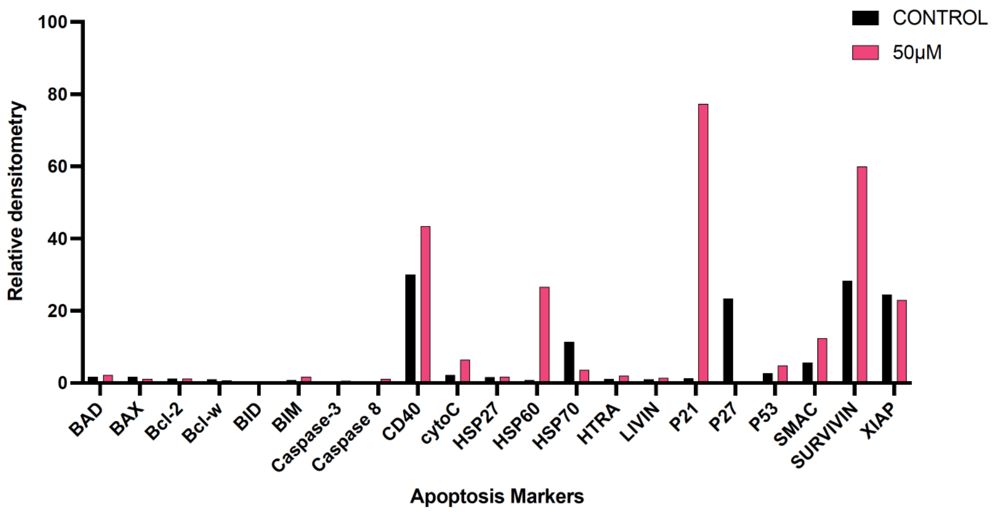

Figure S5. RK-10 Induces p21 Expression. MDA-MB 231 cells were cultured and treated with 0 or 50 µM of RK-10 for 24 hours. Graph depicts the normalized mean pixel density of protein levels of lysates prepared from the MDA-MB 231 cells using the Human Apoptosis Antibody Array Membrane where various antibodies were spotted in duplicate, including 4 positive and 3 negative controls and 1 blank, Array signals were analyzed using Image Lab (BioRad) software. Values from duplicate spots were averaged and plotted. Results are expressed as the mean unit.
